# ThLaeA functions as a conditional repressor to maintain metabolic homeostasis in *Trichoderma hypoxylon*

**DOI:** 10.1128/mbio.00189-26

**Published:** 2026-05-13

**Authors:** Wei Li, Zili Song, Huan Liu, Huomiao Ran, Wenzhao Wang, Kuan Li, Nancy P. Keller, Wen-Bing Yin

**Affiliations:** 1 State Key Laboratory of Microbial Diversity and Innovative Utilization, Institute of Microbiology, Chinese Academy of Sciences, Beijing, China; 2 College of Agriculture and Biology, Liaocheng University 58291, Liaocheng, China; 3 Department of Medical Microbiology and Immunology, University of Wisconsin-Madison, Madison, USA; 4 Medical School, University of Chinese Academy of Sciences 74519, Beijing, China; Universidade de Sao Paulo Campus de Ribeirao Preto, Ribeirao Preto, Sao Paulo, Brazil

**Keywords:** LaeA, secondary metabolism, metabolomic–transcriptomic integration, regulatory network, filamentous fungi

## Abstract

The global regulator LaeA is widely recognized as a master activator of secondary metabolism and development in filamentous fungi. Yet its role under genetically buffered or metabolically stable conditions—where canonical phenotypes are masked—remains poorly understood. This study aimed to characterize the LaeA ortholog (*ThlaeA*) in *Trichoderma hypoxylon* and to elucidate its regulatory role in a Δ*Thtri5* background, where trichodiene synthase function is disrupted. We constructed single and double knockout mutants (Δ*ThlaeA*, Δ*Thtri5*, and Δ*ThlaeA*Δ*Thtri5*) and performed integrated metabolomic and transcriptomic analyses to assess global regulatory effects. Additional assays evaluated oxidative stress responses and biocontrol activity. The deletion of *ThlaeA* alone had negligible effects on secondary metabolite production, whereas disruption of *ThlaeA* in the Δ*Thtri5* background restored biosynthesis of major terpenoid compounds abolished in Δ*Thtri5*. Metabolomics revealed that *ThlaeA* regulates 48.4% of metabolites in the wild-type but 31.3% in Δ*Thtri5*, while transcriptomics showed restoration of 16.7% of gene expression dysregulated in Δ*Thtri5*. Phenotypically, *ThlaeA* deletion reinstated oxidative stress sensitivity and partially attenuated biocontrol efficacy. *ThlaeA* acts as a conditional repressor that counterbalances Thtri5-dependent perturbations to maintain metabolic homeostasis. This work redefines the LaeA paradigm and provides a framework for understanding the context-dependent regulation of secondary metabolism in fungi.

**IMPORTANCE:**

LaeA plays compelling roles in secondary metabolism and development in filamentous fungi. However, related research also found that genetic operations of LaeA have no obvious effect on the metabolic spectrum in some fungal species. Many attempts have been made to decipher the phenomenon and to explain how about the function of LaeA in these cases. Here, we identified a ThlaeA in *Trichoderma hypoxylon*. The deletion of ThlaeA alone did not alter secondary metabolism but restored metabolite production in a Δ*Thtri5* background. Integrated metabolomic and transcriptomic analyses revealed that ThlaeA modulates metabolic homeostasis by compensating for Thtri5-related perturbations. ThlaeA-Thtri5 interaction regulates oxidative stress responses and membrane transport pathways, coupling secondary metabolism with physiological adaptation. This regulatory model broadens the understanding of the LaeA protein family. Fine-tuning this pathway can enhance the environmental adaptability and agricultural biocontrol potential of *Trichoderma* strains, while boosting bioactive secondary metabolite production and optimizing fungal cell factories. This study advances fundamental insights into fungal metabolic regulation and provides a rational basis for strain improvement and biotechnological applications in agriculture and industry.

## INTRODUCTION

Fungal secondary metabolites (SMs) represent invaluable resources for pharmaceutical discovery and play critical roles in fungal ecological adaptation (1, 2). These compounds are typically encoded by biosynthetic gene clusters (BGCs) in fungal genomes. However, a substantial proportion of BGCs remain silent or expressed at low levels under laboratory conditions, impeding the exploration of fungal natural products. These silent BGCs are under strict regulatory control, remaining repressed until appropriate environmental signals trigger their expression (3, 4). Understanding these transcriptional mechanisms, therefore, represents a pressing need in the field. To date, fungal regulatory systems include pathway-specific regulators, global regulators, and epigenetic modifiers, all of which coordinately control metabolic and developmental processes (3). Among them, global regulators can simultaneously activate or repress multiple BGCs, leading to extensive rewiring of metabolic outputs. Consequently, they are regarded as powerful tools for discovering novel SMs and elucidating developmental mechanisms in filamentous fungi.

LaeA stands as one of the most extensively studied global regulators in filamentous fungi. Initially identified in *Aspergillus nidulans*, it upregulates the production of several SMs, including sterigmatocystin, penicillin, and lovastatin. Its expression is negatively regulated by the Zn₂Cys₆ transcription factor AflR, with additional modulation by protein kinase A and RasA signaling pathways (5). Subsequent research has established LaeA as a key regulator of morphogenesis, virulence, and the expression of cryptic BGCs across diverse Ascomycetes, including *Aspergillus*, *Penicillium*, and *Fusarium* species (6–10). LaeA functions within the “Velvet Complex” alongside VelA and VelB to coordinate secondary metabolism and fungal development (2) and also regulates transcription through heterochromatin reorganization via chromatin-mediated mechanisms (11). In most documented cases, LaeA acts as a positive regulator of SM production. For instance, its deletion reduces or abolishes synthesis of penicillin and lovastatin in *A. nidulans* (12), ochratoxin A in *Aspergillus carbonarius* (13) and *Aspergillus ochraceus* (14), aflatoxins in *Aspergillus flavus* (15), and various other metabolites in related species such as *Aspergillus niger* (16), *Aspergillus pachycristatus* (17), *Alternaria alternata* (18, 19), *Fusarium verticillioides* (20), and *Penicillium expansum* (21, 22). Conversely, LaeA overexpression has enabled discovery of new bioactive compounds in several endophytic fungi (23–26). However, emerging evidence indicates that LaeA can also repress SM biosynthesis under specific contexts: its disruption enhances the production of dothistromin in *Dothistroma septosporum* (27) and activates synthesis of novel piperazine derivatives in *Aspergillus flavipes* (28). In *Magnaporthe oryzae*, MoLAEA negatively regulates sporulation and melanization while increasing penicillin G production upon overexpression (29).

LaeA’s regulatory influence extends beyond direct BGC control to encompass broader network interactions with other regulators. In *Penicillium oxalicum*, both LaeA and the transcription factor CreA are required for normal asexual development and SM cluster expression, with CreA deficiency partially compensating for metabolic defects in Δ*laeA* mutant (30). In *Aspergillus fumigatus*, LaeA and the sporulation regulator BrlA are central components of a network governing tissue-specific metabolism (31), while the bZIP protein MetR and the transcription factor NosA can, respectively, rescue selenite sensitivity and germination defects in a Δ*laeA* mutant (32, 33). Notably, the deletion of *laeA* in *Botrytis cinerea* produces no obvious metabolic or developmental phenotypes (34), suggesting context-dependent functional divergence and underscoring the need for nuanced, species-specific investigations.

*Trichoderma hypoxylon*, a fungicolous fungus, produces diverse SMs including trichothecenes, sesquiterpenoids, aspergillazines, and tricinoloniol acids (35–39). In previous work, we isolated *T. hypoxylon* CGMCC 3.17,906 from the stroma of *Hypoxylon anthochroum* (38) and established an efficient genetic platform by disrupting orthologs of *ku70* and *lig4* (40). Through genetic dereplication, we demonstrated that the trichothecene synthase gene *Thtri5* was essential for the production of terpene-derived SMs. Its disruption abolished synthesis of major SMs—tricinoloniol acids A–C, fusidilactone A, and harzianum B—and downregulated the terpene cyclase gene *ThtraA* (37). The Δ*Thtri5* mutant, thus, provided a metabolically simplified background ideal for functional genetic studies.

Here, we identify the LaeA ortholog ThlaeA in *T. hypoxylon* and demonstrate its role as a negative regulator of tricinoloniol acids A–C and fusidilactone A production. Remarkably, we find that ThlaeA deletion restores SM biosynthesis and gene expression in the Δ*Thtri5* background, as confirmed through metabolic profiling, transcriptomics, and metabolomics. Furthermore, the deletion of ThlaeA partially restores the oxidative stress response in the Δ*Thtri5* mutant, while attenuating its biocontrol efficacy. Our findings provide novel insights into the context-dependent regulatory mechanisms of LaeA in filamentous fungi.

## RESULTS

### Identification of ThlaeA and its conditional regulatory role in secondary metabolism

Our investigation began with the comprehensive characterization of LaeA orthologs in *T. hypoxylon* CGMCC 3.17,906. Through systematic genome mining, we identified AXL154_06481, designated ThlaeA, which encodes a protein exhibiting 77.55% and 77.11% identity with characterized LAE1 proteins from *T. atroviride* and *T. reesei*, respectively. The encoding proteins of the candidate showed sequence identity of 36.30% with LaeA from *A. nidulans* at the amino acid level (Tables S3 to S5) (41, 42). The cDNA sequence of *ThlaeA* is 1,032 bp in length and encodes a protein of 344 amino acids. A total of 246 ThlaeA homologs was conducted by clustering analysis. The result showed that fungal LaeA family mainly gathered in two clades, the clade including ThlaeA was separated with the well-known positive regulator LaeA from *A. nidulans*, indicating its different regulatory function (Fig. S1A). The homologs in this clade are widely distributed in four classes, that is, Eurotiomycetes (6.4%), Dothideomycetes (17.0%), Leotiomycetes (29.8%), and Sordariomycetes (46.8%). Phylogenetic analysis revealed that ThlaeA clusters within a distinct evolutionary lineage separate from the canonical *A. nidulans* LaeA (Fig. S1B), suggesting potential functional specialization in this fungicolous species.

**Fig 1:**
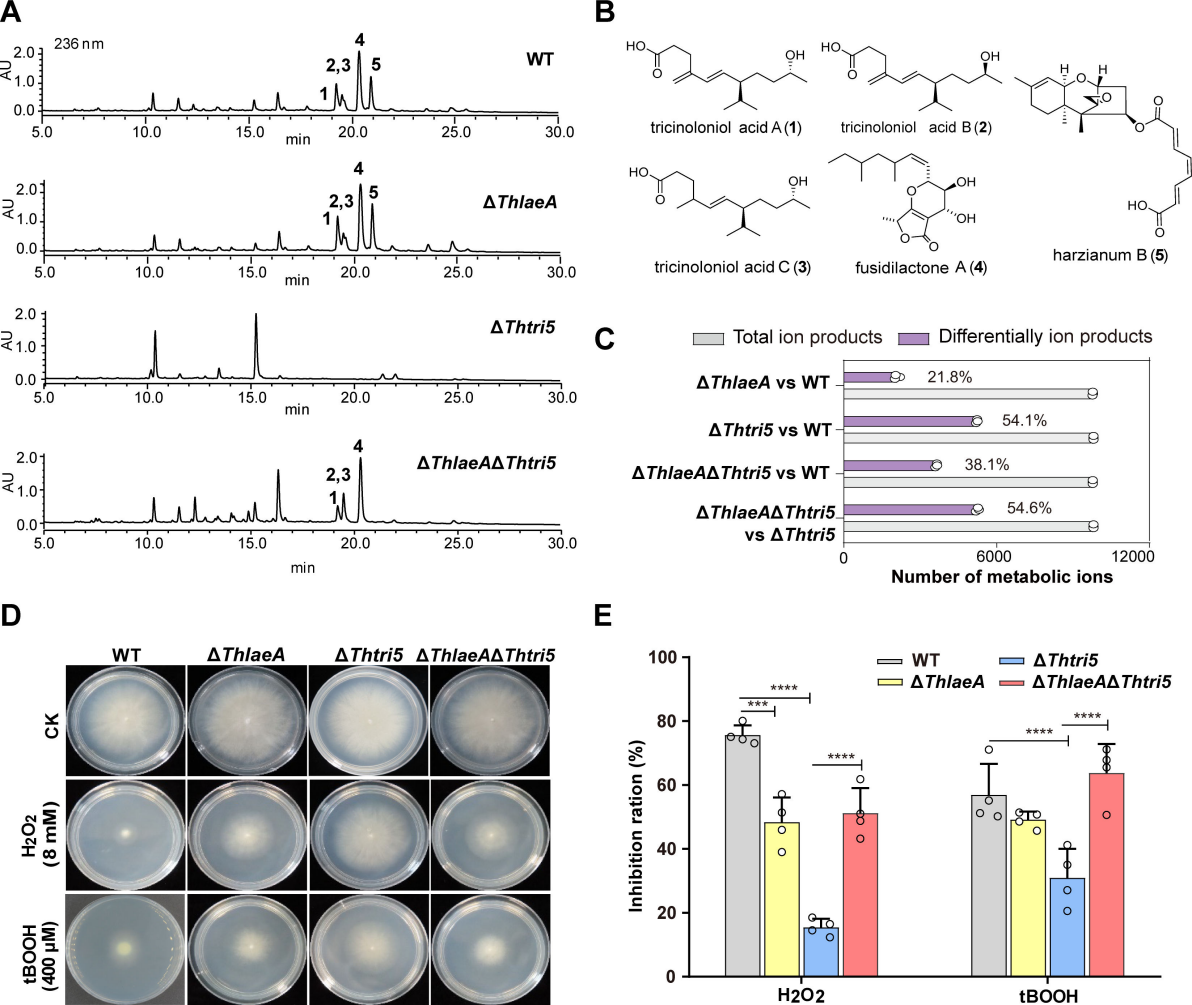
ThlaeA compensates for the secondary metabolic alterations and oxidative stress resistance induced by Thtri5 in *T. hypoxylon*. (**A**) Metabolic profiling of *T. hypoxylon* and its mutant strains. UV absorptions at 236 nm are illustrated. (**B**) Structures of representative secondary metabolites **1**–**5** . (**C**) Global metabolic ion product changes regulated by ThlaeA and Thtri5. |Log_2_fold change| > 1 and −log_10_(*P*-value) > 1.3 indicate a significant difference. (**D**) Mycelia growth of *T. hypoxylon* and its mutants under different oxidative stresses. (**E**) The inhibition ratio of H_2_O_2_ and tBOOH on *T. hypoxylon* and its mutants. All strains were grown on PDA at 25°C for 4 days. All error bars are expressed as mean ± SD. Statistical analysis was performed by using One-way ANOVA (significant ***P < 0.001, ****P < 0.0001).

To elucidate ThlaeA’s regulatory function, we generated *ThlaeA* deletion and overexpression strains (Δ*ThlaeA* and *OE::ThlaeA*) and conducted thorough metabolic profiling (Fig. S2 and S3) (37). Strikingly, HPLC analysis revealed that the secondary metabolite (SM) profile of the Δ*ThlaeA* mutant grown on rice medium was nearly identical to wild-type strain, whereas *ThlaeA* overexpression strongly inhibited the production of major metabolites, including tricinoloniol acids A–C (**1**-**3**), fusidilactone A (**4**), and harzianum B (**5**) (Fig. 1A and Fig. S4 to S6). As these metabolites are also positively regulated by Thtri5, a functional link between Thtri5 and ThlaeA is implicated. We found that the functional role of *ThlaeA* deletion became clear only in the *Thtri5* deletion background. While Δ*Thtri5* single mutants completely lacked production of tricinoloniol acids A–C (**1**-**3**), fusidilactone A (**4**), and harzianum B (**5**) (37, 40), the Δ*ThlaeA*Δ*Thtri5* double mutant exhibited remarkable restoration of these metabolites (Fig. 1B). This genetic suppression phenomenon was further validated through reciprocal experiments showing that *ThlaeA* overexpression in either wild-type or Δ*Thtri5* backgrounds strongly suppressed metabolite production (Fig. S4 to S10). These results indicated that the biosynthetic genes involved in these SMs in Δ*Thtri5* mutant were partly reactivated by the absence of *ThlaeA*.

Further statistical analysis of metabolic ion products from different wild-type strains and mutant strains revealed that, among the 9,830 detected metabolic ions, differential abundance analysis revealed that Δ*Thtri5* mutants exhibited the most severe metabolic alterations, with 5,316 ions (54.1%) significantly changed compared to wild-type (Fig. 1C; Table S6). In contrast, Δ*ThlaeA* single mutants showed relatively modest changes affecting 2,143 ions (21.8%). The double Δ*ThlaeA*Δ*Thtri5* mutant displayed an intermediate phenotype with 3,745 altered ions (38.1%) (|log_2_fold change| > 1 and –log_10_ (*P*-value) > 1.3), representing significant metabolic recovery (Fig. 1C; Table S6).

### ThlaeA modulates oxidative stress responses and biocontrol ability through conditional genetic interactions

Given the established connections between secondary metabolism and oxidative stress response, we systematically evaluated stress sensitivity across all mutants to the peroxidation-inducing agents H_2_O_2_ (8 mM) and tBOOH (400 μM), respectively. *T. hypoxylon* wild-type strain exhibited differential sensitivity to H_₂_O_₂_ (75.7% inhibition) and tBOOH (36.5% inhibition), which suggested distinct antioxidant mechanisms for different oxidative challenges (Fig. 1D through E). Compared with the wild-type, the Δ*ThlaeA* mutant showed a significant decrease in sensitivity to H_₂_O_₂_ and tBOOH (with the inhibitory effects reduced to 48.4% and 35.5%, respectively). However, *Thtri5* deletion resulted in weaker sensitivity to H_₂_O_₂_ and tBOOH, with inhibitory effects of 15.6% and 28.25%, respectively. Most remarkably, in the Δ*Thtri5* background, additional *ThlaeA* deletion significantly increased sensitivity to both oxidants, with H_₂_O_₂_ inhibition rising to 51.2% and tBOOH to 59.5% compared to Δ*Thtri5* alone, respectively (Fig. 1D through E). The above indicates that *ThlaeA* deletion in the Δ*Thtri5* background increases its sensitivity to oxidative stress induced by both H_₂_O_₂_ and tBOOH and further imply that ThlaeA regulates physiological functions related to oxidative stress either independently or conditionally in *T. hypoxylon*.

In nature, *T. hypoxylon* is a fungicolous strain obtained from *Hypoxylon* sp. and is capable of competitively growing against its host (38). The conditional nature of ThlaeA’s regulatory function was further evidenced in ecological competition assays. Confrontation experiments with *Hypoxylon* sp. H2607 revealed that wild-type of *T. hypoxylon* exhibited 54.9% inhibition, while single deletions of *ThlaeA* or *Thtri5* reduced inhibition to 50% and 40%, respectively. In contrast, the Δ*ThlaeA*Δ*Thtri5* double mutant exhibited an inhibition rate of only 16%. These results indicate that ThlaeA and Thtri5 regulate the antagonistic activity of *T. hypoxylon* against pathogenic fungi through additional mechanisms (Fig. S11A and B). Collectively, demonstrating that the ThlaeA-Thtri5 interaction critically governs fungal antagonistic capacity.

### Global metabolomic profiling reveals extensive metabolic restoration by ThlaeA deletion

**Fig 2:**
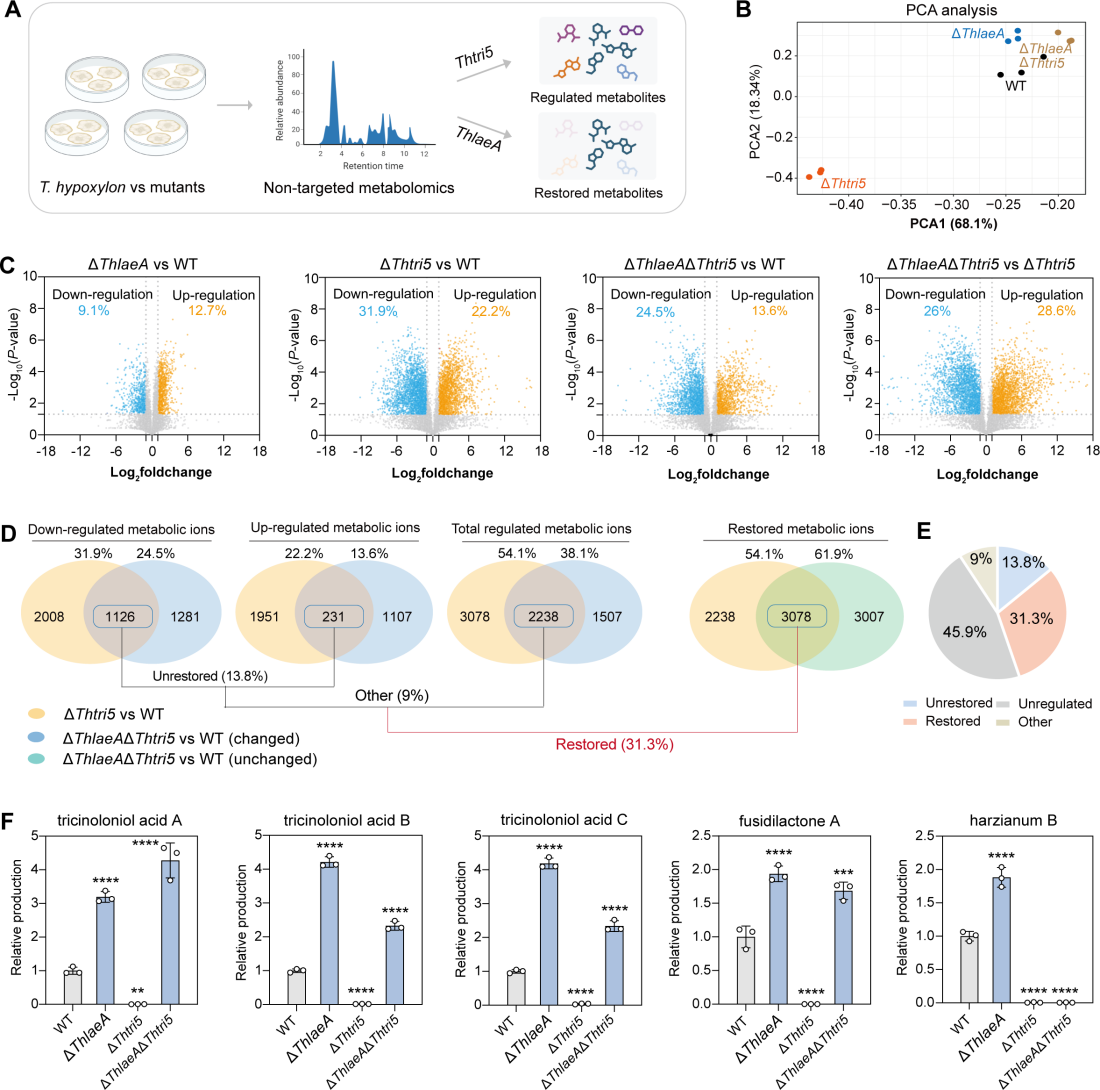
Metabolomic analysis of ThlaeA and Thtri5-induced global metabolite changes. (**A**) Non-targeted metabolomics analysis workflow of *T. hypoxylon* and its mutants. (**B**) Principal component analysis of total ion products in *T. hypoxylon* and its mutants. (**C**) Volcano plots showing the differentially regulated metabolic ion products in *T. hypoxylon* and its mutants. |Log_2_fold change| > 1 and −log_10_(*P*-value) > 1.3 indicate a significant difference. (**D**) Venn diagram showing metabolic ion products restored by ThlaeA in Δ*Thtri5* background mutant. (**E**) Overview of metabolic ion products coordinated by ThlaeA and Thtri5. (**F**) Representative secondary metabolites coordinated by ThlaeA and Thtri5. All error bars are expressed as mean ± SD. Statistical analysis was performed by using one-way ANOVA (significant at **P < 0.01, ***P < 0.001, ****P < 0.0001).

To obtain a systems-level understanding of ThlaeA’s regulatory impact in fungal metabolism, we conducted comparative metabolomics analysis of all mutants and the wild-type strain (Fig. 2A). Principal component analysis clearly separated the metabolic profiles of different genotypes, with Δ*Thtri5* and Δ*ThlaeA*Δ*Thtri5* forming distinct clusters from wild-type and Δ*ThlaeA* (Fig. 2B). This global separation underscores the profound metabolic disruption caused by Thtri5 deletion and the substantial restoration achieved through additional ThlaeA disruption. Specifically, the Δ*ThlaeA* mutant showed only minor changes in differential ionic products, with 899 (9.1%) downregulated and 1,244 (12.7%) upregulated (Fig. 2C). In contrast, the Δ*Thtri5* mutant exhibited more extensive changes in differential ionic products, including 3,134 (31.9%) downregulated and 2,182 (22.2%) upregulated (Fig. 2C). Interestingly, the Δ*ThlaeA*Δ*Thtri5* mutant displayed moderate changes in differential ionic products, with 2,407 (24.5%) downregulated and 1,338 (13.6%) upregulated (Fig. 2C). Further comparison of metabolite ion abundances between the Δ*ThlaeA*Δ*Thtri5* double mutant and Δ*Thtri5* single mutant revealed 2,559 (26%) downregulated and 2,806 (28.6%) upregulated (Fig. 2C). These findings demonstrate that the absence of ThlaeA partially reverses the metabolic alterations induced by Thtri5 deficiency.

Detailed comparison demonstrated that 5,316 metabolite ions altered in the Δ*Thtri5* mutant (representing 54.1% of the total), 3,078 (31.3%) were restored after *ThlaeA* deletion, 1,357 (13.8%) remained unrestored, and 881 (9%) exhibited distinct regulatory patterns (Fig. 2D and E; Table S6). Notably, the previously characterized compounds **1-4** that were absent in the Δ*Thtri5* mutant were reactivated following additional ThlaeA deletion. Since the harzianum B synthesis was directly controlled by Thtri5, it remained unactivated even after ThlaeA deletion (Fig. 2F). Collectively, these results demonstrate that the presence of ThlaeA inhibits the recovery of metabolite ions in the Thtri5-deleted strain, which can be explained by ThlaeA negatively regulating Thtri5-mediated global metabolic changes—this inhibitory effect is only abrogated when LaeA was also deleted.

### Transcriptomic analysis uncovers ThlaeA’s cryptic regulatory network

RNA-seq analysis provided mechanistic insights into ThlaeA’s conditional regulatory functions on the secondary metabolism, oxidative stress, and ecological competition (Fig. S12; Table S7). Through comparative transcriptomic analysis of the wild-type, Δ*ThlaeA,* Δ*Thtri5*, and Δ*ThlaeA*Δ*Thtri5* strains, 11,542 unique transcripts were detected, with hierarchical clustering clearly separating Δ*Thtri5* from other genotypes while grouping Δ*ThlaeA* with wild-type (Fig. S12A). Heatmap analysis revealed that compared with the wild-type strain, the Δ*Thtri5* mutant exhibited a large number of differentially expressed genes (DEGs). In contrast, the gene expression trend of the Δ*ThlaeA* mutant was consistent with the wild-type strain (Fig. S12B). This led us to speculate that the deletion of *ThlaeA* may remodulate the altered gene expression in the Δ*Thtri5* mutant, potentially restoring it to wild-type level. To confirm this hypothesis, a volcano plot analysis of the transcriptome was performed to evaluate the upregulation or downregulation of these DEGs (Fig. S12C).

**Fig 3:**
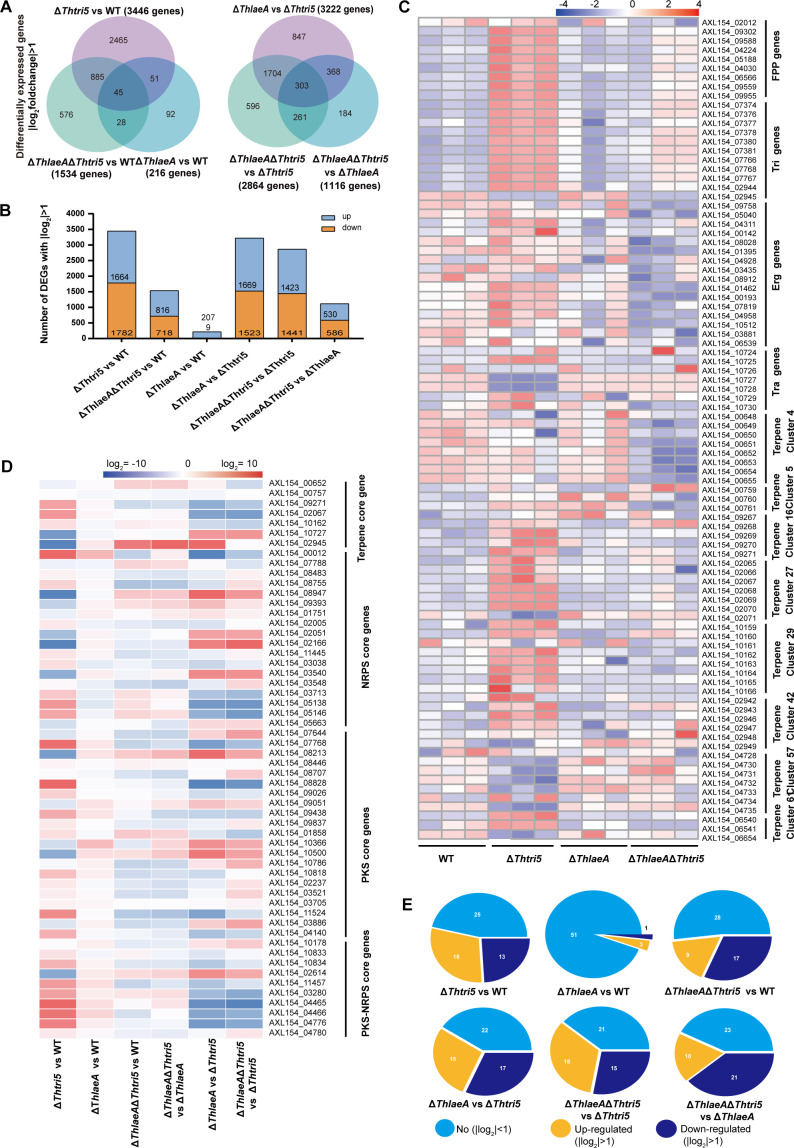
Transcriptome analyses of differentially expressed genes in the Δ*Thtri5* and Δ*ThlaeA* mutants vs the control in *T. hypoxylon*. (**A**) Venn diagram showing the genes co-regulated by the *Thtri5* and *ThlaeA*. (**B**) Number of differentially expressed genes in Δ*Thtri5*, Δ*ThlaeA*, and Δ*ThlaeA*Δ*Thtri5* mutants compared to the control. (**C**) Heatmap showing expression levels of terpenoid synthesis genes in Δ*Thtri5*, Δ*ThlaeA*, and Δ*ThlaeA*Δ*Thtri5* mutants compared to the control. (**D**) Heatmap showing expression levels of other secondary metabolite biosynthesis backbone genes in Δ*Thtri5*, Δ*ThlaeA*, and Δ*ThlaeA*Δ*Thtri5* mutants compared to the control. (**E**) Number of differentially expressed backbone genes involved in secondary metabolite biosynthesis in *T. hypoxylon* and its mutants. Light blue represents the number of gene clusters with no significant changes, orange represents the number of gene clusters with significant upregulation, dark blue represents the number of gene clusters with significant downregulation. Differentially expressed genes: *P*adj < 0.05, |log_2_fold change| > 1.

Compared to the wild-type strain, Δ*ThlaeA* single mutant had only 216 DEGs (1.9% of transcriptome), whereas the Δ*Thtri5* mutant exhibited massive transcriptomic dysregulation affecting 3,446 genes (30%). The double mutant showed 1,534 DEGs (13.3%) compared to wild-type, representing substantial transcriptomic restoration (Fig. 3A and B; Fig. S12C). Among them, 1,664 genes were upregulated and 1,782 genes were downregulated in the Δ*Thtri5* mutant. Direct comparison between Δ*ThlaeA*Δ*Thtri5* and Δ*Thtri5* mutants identified 816 upregulated and 718 downregulated genes in the double-deficient mutant, demonstrating widespread reversal of Thtri5-dependent transcriptional changes (Fig. 3A and B).

Focusing on the biosynthetic gene clusters (BGCs) of SMs, the DEGs related to terpene biosynthesis (log_2_fold change value > 1 and adjusted *P* value < 0.05) were categorized into four classes: farnesyl diphosphate (FPP), ergosterol (Erg), harzianum (Tri), and tricinoloniol acids (Tra) in *T. hypoxylon* (Table S8). Heatmap analysis revealed that disruption of *ThlaeA* has no significant effect on the expression levels of these terpene biosynthetic genes, whereas the *Thtri5* deletion exerted a substantial impact (Fig. 3C). Particularly noteworthy was the reactivation of the tricinoloniol acid synthase gene *traA* (*AXL154_10727*) and associated terpenoid biosynthetic genes in the double-deficient mutant compared to their significant downregulation in the Δ*Thtri5* mutant (Fig. 3C). This transcriptional restoration directly correlates with the observed metabolic recovery and provides molecular evidence for ThlaeA’s repressive role in secondary metabolism.

Expression analysis of 54 BGC backbone genes via transcriptomic data revealed that the DEGs included those encoding polyketide synthases (PKS), non-ribosomal peptide synthases (NRPS), terpene synthases, and hybrid enzymes, indicating that ThlaeA has a broad regulatory scope (Table S9). Disruption of *ThlaeA* resulted in the significant upregulation of only two backbone genes compared to the wild-type strain. In contrast, 23 backbone genes, including seven PKSs, eight NRPSs, five PKS-NRPS hybrids, and three terpene synthases-encoding genes, were significantly upregulated in the Δ*Thtri5* mutant (Fig. 3D). Additionally, 13 backbone genes, comprising 8 PKSs, 3 NRPSs, and 2 terpene synthases-encoding genes, were significantly downregulated in the same mutant. Notably, the expression level of 30 backbone genes was either reversed or compensated to wild-type levels through the deletion of *ThlaeA* in Δ*Thtri5* mutant (Fig. 3D and E). These data demonstrate ThlaeA’s ability to restore regulatory expression across multiple biosynthetic pathways in the Δ*Thtri5* mutant.

### Membrane transport machinery as a notable target of ThlaeA-Thtri5 regulation

**Fig 4:**
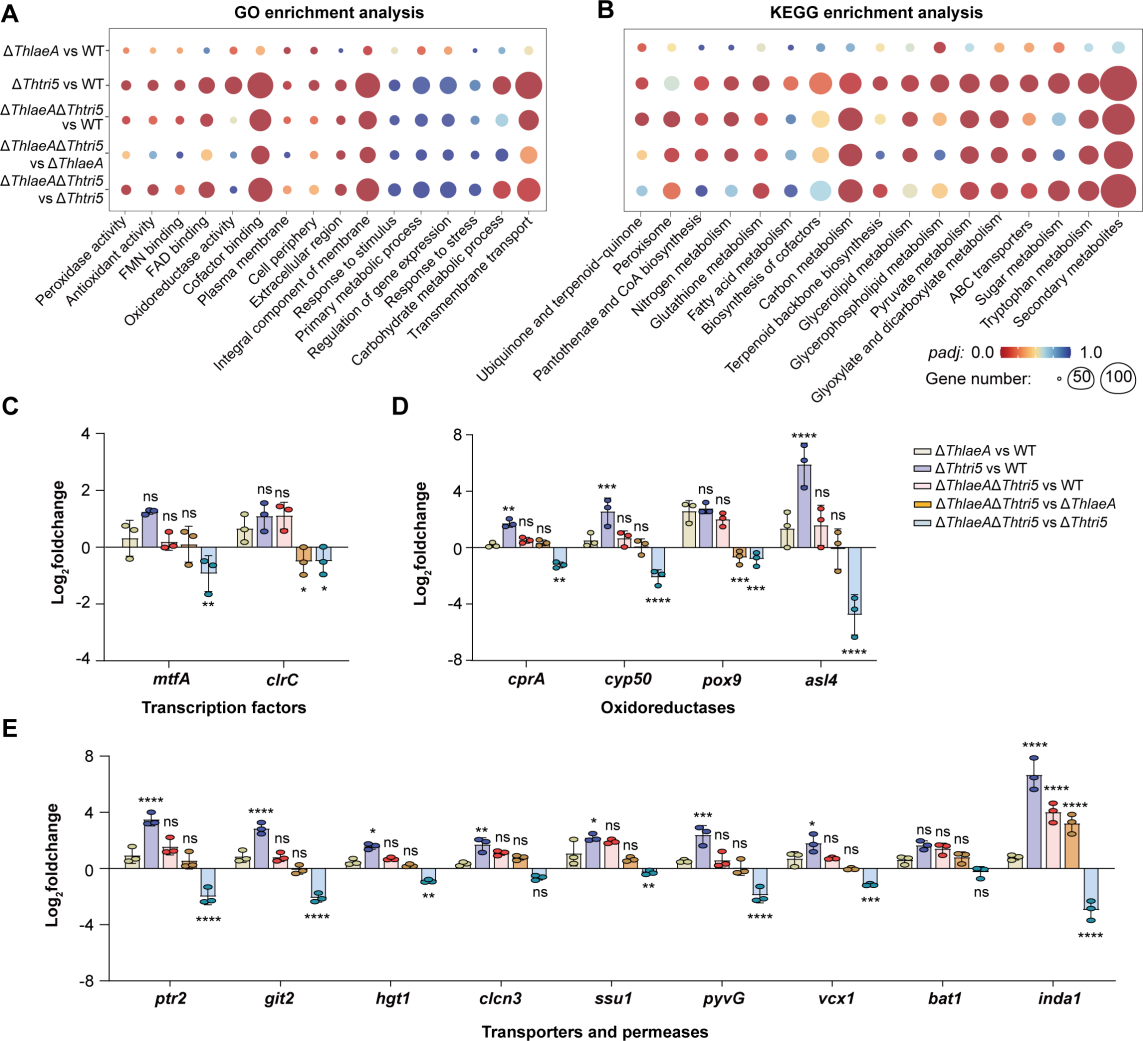
Functional enrichment analysis of differentially expressed genes in mutants compared with the control. (**A**) GO enrichment analysis of genes regulated by Thtri5 and ThlaeA in *T. hypoxylon*. (**B**) KEGG enrichment analysis of genes regulated by Thtri5 and ThlaeA in *T. hypoxylon*. (**C–E**) Expression levels of transcription regulators (TFs) (**C**), oxidoreductases (**D**), and membrane transport (**E**)-related genes in Δ*Thtri5*, Δ*ThlaeA*, and Δ*ThlaeA*Δ*Thtri5* mutants compared to the control. All error bars are expressed as mean ± SD. Statistical analysis was performed by using one-way ANOVA (“ns”: not significant. Significant at *P < 0.05, **P < 0.01, ***P < 0.001, ****P < 0.0001).

GO and KEGG enrichment analyses provided molecular support for the aforementioned changes, showing that genes related to membrane transport and secondary metabolism were significantly enriched in Δ*Thtri5* (Fig. 4A and B; Table S9). As key mediators linking environmental stress to target gene expression, transcription factors (TFs) associated with the enriched pathways include two extra-cluster TFs (*mtfA* and *clrC*) and four intra-cluster TFs (Fig. 4C; Fig. S13; Table S11). Notably, *mtfA*—the homologous gene of *AXL 154_00617*—has been reported to regulate sterigmatocystin biosynthesis by mediating oxidative stress responses in *A. flavus* (43). Similarly, *clrC*, the homolog of *AXL 154_05915*, has also been documented to be associated with oxidative stress in *P. oxalicum* (44). Our transcriptomic analysis revealed that *mtfA* was appropriately upregulated in the Δ*Thtri5* mutant, whereas the additional deletion of *ThlaeA* restored its expression to the level in the wild-type strain (Fig. 4C). This result is consistent with the above conditional regulatory role of ThlaeA in secondary metabolism and oxidative stress, suggesting that *mtfA* is a relevant mediator in the ThlaeA-Thtri5 regulatory network.

Furthermore, the expression analysis of redox-related genes revealed that the cytochrome P450 genes (*cprA* and *cyp50*) and oxidoreductase genes (*pox9* and *asl4*)—which were dysregulated in Δ*Thtri5*—were mostly restored to normal levels in the double mutant (Fig. 4D ; Table S10). This result is consistent with the conditional regulatory role of ThlaeA in oxidative stress. Notably, our transcriptomic analysis revealed that membrane transport-related genes represented the most significantly enriched category among ThlaeA-Thtri5 cross-regulated genes (Fig. 4E). These included comprehensive sets of genes encoding permeases, transmembrane transporters, and secretion system components, all of which were dramatically dysregulated in Δ*Thtri5* but appropriately restored in Δ*ThlaeA*Δ*Thtri5*, which fully demonstrates the important role of the membrane transport machinery in ThlaeA-Thtri5 regulatory function.

The coordinated regulation of transport machinery with biosynthetic genes suggests an integrated regulatory strategy where ThlaeA and Thtri5 collectively control not only metabolite production but also their secretion and potential intercellular signaling functions. This finding expands the functional significance of the ThlaeA-Thtri5 interaction beyond metabolism per-se to encompass cellular communication and environmental interaction.

### Integrated model of ThlaeA-Thtri5 regulatory network in metabolic homeostasis

**Fig 5:**
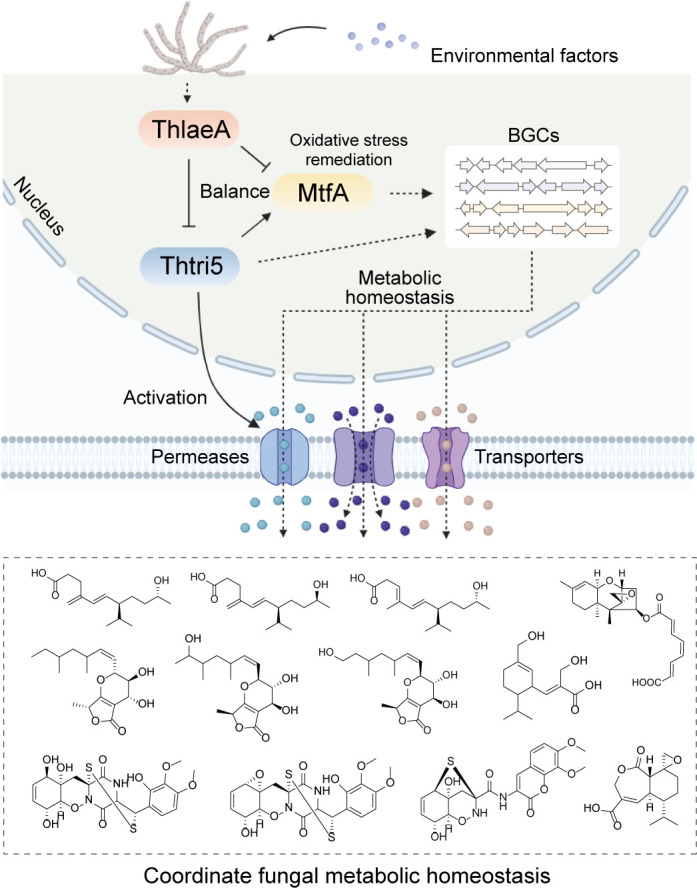
Putative model of metabolic homeostasis maintained by the ThlaeA-Thtri5 regulatory network. Thtri5 functions as a key positive regulator of global secondary metabolism, whereas ThlaeA acts as a negative regulator of this process. Notably, the inhibitory activity of ThlaeA is dependent on the presence of Thtri5, through which it exerts a reciprocal inhibitory effect on Thtri5 function. In response to environmental cues, activated ThlaeA suppresses the biosynthesis of secondary metabolites. Conversely, Thtri5 not only promotes metabolite production but also counteracts LaeA-mediated inhibition, thereby establishing a regulatory balance between the two factors. This dynamic interplay maintains metabolic homeostasis, enabling adaptive responses to environmental fluctuations.

Based on our comprehensive genetic, metabolic, and transcriptomic analyses, we propose a refined model for the ThlaeA-Thtri5 regulatory network in maintaining metabolic homeostasis (Fig. 5). In this model, Thtri5 functions as the primary positive regulator driving SMs production, while ThlaeA serves as a conditional negative modulator whose activity becomes significant only when metabolic balance is disrupted. The mutually antagonistic relationship between ThlaeA and Thtri5 creates a robust homeostatic system that maintains metabolic stability under fluctuating environmental conditions. This regulatory interplay extends to oxidative stress responses and membrane transport processes, creating an integrated network that coordinates metabolic production, stress adaptation, and ecological competition. Our findings demonstrate that ThlaeA’s regulatory significance in *T. hypoxylon* is context-dependent, emerging primarily when the metabolic network experiences perturbation. This conditional functionality explains the cryptic nature of ThlaeA’s regulatory role and provides novel insights into the evolutionary adaptation of global regulatory networks in specialized fungal ecological niches.

## DISCUSSION

Fungi employ sophisticated regulatory systems to activate bioactive SMs synthesis in response to environmental cues. Such regulation occurs at both pathway-specific and global levels. LaeA represents one of the most prominent global regulators in filamentous fungi, with orthologs involved in primary and secondary metabolism, development, and virulence across Ascomycetes (7, 14, 15, 18, 21, 27, 45–48). Our understanding of the regulatory mechanism of LaeA has been steadily expanding (4). Although LaeA is commonly regarded as a positive regulator, its deletion does not always alter SMs profiles in wild-type strains, complicating functional analysis in many species.

In this study, we identified the LaeA ortholog ThlaeA in *T. hypoxylon* and showed that its deletion alone does not enhance SM production, whereas its overexpression suppresses major SMs. In a ∆*Thtri5* background—which lacks major terpenoid SMs and provides a clean metabolic baseline—*ThlaeA* deletion restored the production of compounds **1**–**4** (37). Overexpression of *ThlaeA* in ∆*Thtri5* further suppressed SMs synthesis, reinforcing its repressive role. Metabolomics confirmed that 31.3% of global metabolic alterations in ∆*Thtri5* mutant were reversed by *ThlaeA* deletion, supporting a unique restorative function for ThlaeA in *T. hypoxylon*. Transcriptomic data corroborated this, showing reactivation of the Tra synthase gene *traA* and other terpenoid genes in the double-deficient mutant. ThlaeA, thus, appears to function as a negative regulator and a key restorer of metabolic balance when Thtri5 activity is compromised. Furthermore, the regulatory network between ThlaeA and Thtri5 is intricate. ThlaeA may repress Thtri5 activity indirectly by modulating downstream transcription factors (e.g., MtfA) or cellular stress responses. Direct ThlaeA–Thtri5 interaction to form a complex that coordinately regulates the transcription of secondary metabolite gene clusters is also plausible. This regulatory interplay will be further verified in future studies.

Beyond terpenoid pathways, RNA-seq revealed global transcriptomic restoration in the double-deficient mutant relative to ∆*Thtri5*. Affected genes included PKS, NRPS, terpene, and hybrid backbone genes, indicating broad regulatory influence. In many fungi, BGCs contain pathway-specific TFs that regulate cluster expression (5). Well-known examples include AflR and AflS in aflatoxin biosynthesis (49, 50). LaeA is known to regulate sterigmatocystin clusters via AflR and AflS (7), suggesting that ThlaeA’s restorative function may also operate through TFs. Our identification of restored TFs (including MtfA and ClrC homologs) supports this hypothesis and establishes ThlaeA as a broad-spectrum restorer of regulatory equilibrium.

BGC activation is often linked to environmental stress, particularly oxidative challenge (4). LaeA has been implicated in oxidative stress sensitivity and virulence in several fungi (47). Here, we found that ThlaeA modulates oxidative stress responses differently in wild-type and ∆*Thtri5* backgrounds, with a major role in tBOOH-induced stress. The deletion of *ThlaeA* recalibrated the oxidative stress network in wild-type cells and restored stress sensitivity in ∆*Thtri5 mutant*. We also assessed antifungal capacity via host confrontation assays. Loss of *ThlaeA* reduced virulence toward *Hypoxylon* sp., consistent with reports that LaeA deficiency impairs toxin production and host inhibition in *A. flavus* (15) and in *C. heterostrophus* (51). Thus, reduced antifungal activity in ∆*ThlaeA* mutant likely stems from altered SMs production, particularly of antifungal toxins.

Based on these findings, we propose a model for ThlaeA–Thtri5 interplay in regulating fungal secondary metabolism and environmental adaptation (Fig. 5). In this model, Thtri5 acts as a primary positive regulator of global secondary metabolism, while ThlaeA serves as a negative modulator. ThlaeA’s repressive activity depends on Thtri5, creating a mutually inhibitory relationship. Under environmental stress (e.g., oxidative challenge), activated ThlaeA helps maintain metabolic homeostasis by tuning membrane transport and coordinating oxidative stress responses via TFs like MtfA. Concurrently, Thtri5 not only drives metabolite production but also buffers LaeA-mediated repression, establishing a dynamic balance. This intricate cross-regulation reflects fungal adaptation to complex environments and offers fresh perspectives on the evolution of metabolic plasticity in diverse ecological niches.

Finally, we note that while LaeA and its homolog Lae1 are extensively studied for their central roles in regulating secondary metabolism in other filamentous fungi, research on Lae1 in *Trichoderma* species has historically emphasized its involvement in primary metabolism and development rather than broad secondary metabolite control. First identified in *T. reesei*, Lae1 deletion primarily impairs cellulase gene expression and general fitness (42, 52), with minor effects on BGC expression (53). Lae1 also regulates cellulolytic enzymes in *T. harzianum* (54) and is critical for mycoparasitism in *T. atroviride*: its loss reduces parasitism and the expression of mycoparasitism-upregulated genes (41), and further studies link it to phospholipid metabolism and specific SMs in this species (55). In *T. longibrachiatum*, Lae1 deletion decreases peptaibol production (56), while in *T. asperellum*, Lae1 is associated with salt tolerance (57). Collectively, these findings highlight the need to further investigate whether the impact of ThlaeA on secondary metabolism in *T. hypoxylon* is linked to its roles in primary metabolic processes.

In summary, we identified and characterized ThlaeA, a LaeA ortholog in *T. hypoxylon*, revealing its role as a negative regulator activated only upon *Thtri5* loss. The deletion of *ThlaeA* in the Δ*Thtri5* mutant restored terpene production and reestablished regulatory control over secondary metabolic networks. ThlaeA also modulated oxidative stress responses and ecological adaptability, contributing to physiological homeostasis under environmental stimuli. Our work elucidates how the ThlaeA–Thtri5 network maintains metabolic balance in *T. hypoxylon* and provides a new framework for uncovering cryptic biosynthetic potential and regulatory behaviors in fungi—whether in engineered mutants or under specific environmental conditions.

## MATERIALS AND METHODS

### Strain cultivation

The fungal strains used in this study are detailed in Table S1. The wild-type strain *T. hypoxylon* CGMCC 3.17906 was deposited at the China General Microbiological Culture Collection Center, and its mutant derivatives were cultivated on potato dextrose agar (PDA; BD Difco) at 25°C for growth and SM detection. When necessary, appropriate antibiotics were included. *Escherichia coli* DH5α was utilized for vector construction and DNA propagation. These bacteria were grown at 37°C on Luria-Bertani (LB) medium, composed of 1% NaCl, 1% tryptone, and 0.5% yeast extract, supplemented with ampicillin (50 µg/mL) for selective enrichment.

### *In silico* analysis of ThlaeA and its orthologs

The amino acid sequences of LaeA from *A. nidulans* FGSC A4 (Q6TLK5.1), *T. atroviride* ATCC 74058 (AGT59504.1) and *T. reesei* QM6a (AFK30952.1) and *T. ressei* QM6a were used as queries. The basic local alignment search tool algorithm was used to search LaeA from the database in the National Center for Biotechnology Information resources (NCBI) (https://blast.ncbi.nlm.nih.gov/Blast.cgi). All the LaeA orthologs sequences were analyzed using EFI-EST (https://efi.igb.illinois.edu/efi-est/) and Cytoscape (http://www.cytoscape.org/). The amino acid sequences of ThlaeA with the known LaeA orthologs were aligned using ClustalW. The phylogenetic tree was created with the maximum likelihood method by MEGA7 based on ClustalW multiple alignments. The relative distance of the orthologous proteins was calculated and modified using the Interactive Tree of Life (ITOL) method.

### Plasmid construction and genetic manipulation

The plasmids and oligonucleotide sequences used in this study are listed in Tables S1 and S2, respectively. High-fidelity thermostable DNA polymerase from TransStart FastPfu DNA polymerase (Transgen Biotech, China) was used to amplify the gene fragments, and DNA polymerase from the 2× Taq Mix kit (Tiangen Biotech, China) was used to verify the positive mutants. All PCR amplifications were carried out by a T100 Thermal cycler (Bio-Rad). For gene disruption, approximately 1.4-kb upstream and 1.4-kb downstream fragments of the target genes were amplified from *T. hypoxylon* gDNA and induced into the selected marker gene hygromycin B (*hph*) by the quick-change method according to the protocol in the literature (58).

For *ThlaeA* overexpression, the 1.4-kb 5′ flank of *ThlaeA* promoter sequences and the 1.4-kb of the intact *ThlaeA* open reading frame were amplified using the primers listed in Table S2. The upstream and downstream regions of *ThlaeA* were inserted into pYWL156, which contains the selected marker *hph* and the *gpdA* promoter, to generate the *ThlaeA* overexpression cassette pYHL94. Both the deletion and overexpression cassettes were purified using E.Z.N.A. Cycle-Pure kit (D6492-02, Omega, USA) for protoplast transformation.

### Transformation of *T. hypoxylon*

The transformation of *T. hypoxylon* was performed via polyethylene glycol (PEG)-mediated protoplast transformation as described (38). The single colonies of transformation were picked up and screened on selection PDA plates with appropriate antibiotics (60 μg/mL hygromycin B) after two generations and then verified by diagnostic PCR. For Δ*ThlaeA* mutant verification, the transformants were verified using three pairs of designated primers: *LaeA*-SCR F/*hph*-SCR R, *hp-*SCR F/*LaeA*-SCR R, and *LaeA*-RT F/R (Table S2). Primers of *LaeA*-SCR F/OE -SCR R, *hph-*SCR F/*LaeA*-SCR R, and *LaeA*-RT F/R were used for the verification of mutants of overexpression *ThlaeA* (Table S2).

### RNA isolation and transcriptome sequencing

The positive mutants and wild-type strain were grown on rice medium with appropriate antibiotics at 25°C for 1 week. Total mRNA from mycelia was extracted with TriZol reagent (catalog no. R1000, Lablead Inc., China) according to the manual. Three biological replicates were generated for each sequenced strain. The RNA purity was checked using a KaiaoK5500 Spectrophotometer (Kaiao, Beijing, China). RNA integrity and concentration were assessed using the RNA Nano 6000 Assay Kit of the Bioanalyzer 2100 system (Agilent Technologies, CA, USA). For library preparation, a total amount of 2 μg RNA per sample was used as input material for the RNA sample preparations. Sequencing libraries were generated using the NEBNext Ultra RNA Library Prep Kit for Illumina (#E7530 L, NEB, USA) following the manufacturer’s recommendations. The index codes were added to attribute sequences to each sample.

The raw reads were obtained by transforming the raw image base using Illumina (https://www.illumina.com/) base calling software. Afterward, the adapters and the low-quality reads with more than 5% of the unknown bases were removed to generate clean reads. The clean reads were mapped to the corresponding sequence using TopHat2 software (v2.1.1). The differentially expressed genes were calculated by DESeq2.

### Gene expression analysis

The different gene expression levels were identified with a *P* value < 0.05 and a |log_2_fold change| > 1 between two samples. In addition, we used an automated assignment against the protein domain database KEGG (https://www.kegg.jp/kegg/genome/) and GO (http://omicslab.genetics.ac.cn/GOEAST/tools.php). KEGG analysis was followed by a hypergeometric distribution analysis and a false discovery rate (FDR) method to yield an adjusted *P* value (*P* = 0.05 as a threshold value). The redundant terms were subsequently removed and resulted in significantly enriched KEGG terms. Heatmaps were generated with the differentially expressed genes.

### Metabolomics analysis

The mutant and wild-type strains of *T. hypoxylon* were cultivated on rice medium at 25°C for 7 days, with three biological replicates set up for each group. The metabolites were extracted using 30 mL of ethyl acetate, and the resulting extracts were concentrated to dryness under reduced pressure. The crude extracts were re-dissolved in 1 mL of methanol and then analyzed via liquid chromatography-high resolution mass spectrometry (LC-HRMS) equipped with an ODS column (C18, 250 × 4.6 mm, Waters XTERRA, 5 μm) at a flow rate of 1 mL/min. The methanol and 0.1% formic acid aqueous solution as mobile phases with the following gradient elution program: 10%–30% methanol for 10 min, 30%–70% methanol for 30 min, 70%–90% methanol for 10 min, 100% methanol for 10 min, and re-equilibration to 10% methanol for 5 min. The mass spectrometry data were acquired using Qualitative Analysis B.06.00 software and then converted into a format containing retention time, *m/z* values, and ion peak intensity. Differential analysis was performed using XCMS software, and differentially metabolites were screened by setting the thresholds of |log_2_fold change| > 1 and –log_10_ (*P*-value) > 1.3.

### Growth and development analysis of *T. hypoxylon* strains

To evaluate the stress sensitivity, oxidative stress experiments with different concentrations of oxidative reagents were carried out. The colony diameters are the indicators for condition optimization. The strain was inoculated on PDA media supplemented with the following stress-generating agents: tert-butylhydroperoxide (tBOOH, 0.1 mM, 0.2 mM, 0.3 mM, 0.4 mM, and 0.5 mM) and H_2_O_2_ (1 mM, 3 mM, 5 mM, 7 mM, and 9 mM). All stress plates were incubated at 25°C for 4 days. The appropriate concentrations of tBOOH (0.4 mM) and H_2_O_2_ (7 mM) were used for the stress sensitivity test. All treatments were induced for three replicates.

To test influences on the infection ability of *T. hypoxylon* mutants against host fungi *Hypoxylon* sp. H2607, the tested strains were grown on PDA medium at 25°C for 4 days, and the inhibition ratios were measured with three replicates. Error bars represent the standard deviations. Asterisks represent significant differences compared to the control. All experiments were performed in three independent biological replicates. The mean data were compared for significant differences via the Tukey‒Kramer multiple comparison test by using GraphPad Prism Version 6 software.

### HPLC and LC‒MS analysis of SMs

Analysis of SMs was performed on a Waters HPLC system (Waters 2998, Photodiode Array Detector) with an XTerra MS C18 column (250 × 4.6 mm, 5 μm, Waters). Water (A) and acetonitrile (B), both with 0.1% (vol/vol) formic acid, were used as solvents at a flow rate of 1 mL/min. The substances were eluted with a linear gradient from 5% to 100% solvent B over 40 min, washed with 100% (vol/vol) solvent B for 5 min and equilibrated with 5% (vol/vol) solvent B for 5 min. UV absorption at 236 nm is illustrated. LC‒MS analysis was performed on an Agilent HPLC 1200 series system equipped with a single quadrupole mass selective detector and an Agilent 1100LC MSD model G1946D mass spectrometer by using a Venusil XBP C18 column (3.0 × 50 mm, 3 µm, Bonna-Agela Technologies, China). Water (A) with 0.1% (vol/vol) formic acid and acetonitrile (B) were used as the solvents at a flow rate of 0.5 mL/min. The substances were eluted with a linear gradient from 5% to 100% B over 30 min, washed with 100% (vol/vol) solvent B for 5 min, and equilibrated with 5% (vol/vol) solvent B for 10 min. The mass spectrometer was set in electrospray positive ion mode for ionization.

## Data Availability

Relevant data supporting the findings of this study are available in this article and its supplemental files.

## SUPPLEMENTAL MATERIAL

Supplemental figures and tables mbio.00189-26-s0001.pdf Tables S1-4; Figures S1-13.

Table S5 mbio.00189-26-s0002.xlsx Sequence clustering of ThlaeA.

Table S6 mbio.00189-26-s0003.xlsx Metabolomic data analysis.

Table S7 mbio.00189-26-s0004.xlsx Expression level of total genes.

Table S8 mbio.00189-26-s0005.xlsx DEGs contained in terpene.

Table S9 mbio.00189-26-s0006.xlsx DEGs contained in SMs.

Table S10 mbio.00189-26-s0007.xlsx GO and KEGG data.

Table S11 mbio.00189-26-s0008.xlsx TF, oxidoreductase, and membrane transport genes.

ASM does not own the copyrights to Supplemental Material that may be linked to, or accessed through, an article. The authors have granted ASM a non-exclusive, world-wide license to publish the Supplemental Material files. Please contact the corresponding author directly for reuse.
