# Supplementary material for: ThLaeA functions as a conditional repressor to maintain metabolic homeostasis in Trichoderma hypoxylon
Source: mBio. 2026 May 13;17(6):e00189-26. doi: 10.1128/mbio.00189-26 (PMC13251417; doi:10.1128/mbio.00189-26)
Supplement: Supplemental figures and tables — Tables S1-4; Figures S1-13. [file mbio.00189-26-s0001.pdf]

Supporting Information for

ThLaeA functions as a conditional repressor to maintain metabolic homeostasis in *Trichoderma hypoxylon*

Wei Li[1,2,†], Zili Song[1,†], Huan Liu[1,†], Huomiao Ran[1], Wenzhao Wang[1], Kuan Li[4], Nancy P. Keller[3,*], Wen-Bing Yin[1,4,*]

This file contains:

Supplementary Tables

Table S1. Plasmids and strains used in this study.

Table S2. Primers used in this study.

Table S3. LaeA orthologues for phylogenetic tree in the given organisms and their conservative property with LaeA in *Aspergillus nidulans*.

Table S4. LaeA orthologues and their conservative property with LaeA in *Trichoderma reesei* QM6a.

Supplementary Figures

Figure S1. Phylogenetic tree analysis and sequence clustering of ThlaeA orthologues in fungi.

Figure S2. Generation of the *ThlaeA* deletion mutants in *T. hypoxylon* by diagnostic PCR.

Figure S3. Generation of the OE::*ThlaeA* mutants in *T. hypoxylon* by diagnostic PCR.

Figure S4. Metabolic profiling of HPLC analysis for OE::*ThlaeA* mutants in *T. hypoxylon*.

Figure S5. LC−MS analysis of the compounds **1**−**5** in *T. hypoxylon* wild type.

Figure S6. LC−MS analysis of the compounds **1**−**5** in TYHL14 (Δ*ThlaeA* mutant of *T. hypoxylon*).

Figure S7. LC−MS analysis of the compounds **1**−**5** in TYHL26 (Δ*Thtri5* mutant of *T. hypoxylon*).

Figure S8. LC−MS analysis of the compounds **1**−**5** in TYHL49 (Δ*ThlaeA*Δ*Thtri5* mutant of *T. hypoxylon*).

Figure S9. LC−MS analysis of the compounds **1**−**5** in TYHL55 (OE::*ThlaeA* mutant of *T. hypoxylon*).

Figure S10. LC−MS analysis of the compounds **1**−**5** in TYHL56 (OE::*ThlaeA*Δ*Thtri5* mutant of *T. hypoxylon*).

Figure S11. Antagonistic activity of *T. hypoxylon* and its mutants against the pathogenic fungus *Hypoxylon* sp. H2607.

Figure S12. Transcriptome-wide analyses of differentially expressed genes in the Δ*Thtri5* and Δ*ThlaeA* mutants versus the control in *T. hypoxylon*.

Figure S13. Transcriptome analysis of transcription regulators (TFs), oxidoreductases, and membrane transport-related genes in Δ*Thtri5*, Δ*ThlaeA* and Δ*ThlaeA*Δ*Thtri5* mutants compared to the control.

Supplementary References

Supplementary Tables

Table S1. Plasmids and strains used in this study.

Plasmids/Strains	Descriptions	References
WT	*T. hypoxylon* CGMCC 3.17906	(1)
TYYH14	Δ*ThlaeA*::*hph*	This study
TYHL26	Δ*Thtri5*::*neo*	(2)
TYHL49	Δ*ThlaeA*::*hph,* Δ*Thtri5*::*neo*	This study
TYHL55	OE::*ThlaeA*::*hph*	This study
TYHL56	OE::*ThlaeA*::*hph,* Δ*Thtri5*::*neo*	This study
H2607	*Hypoxylon* sp. 2607	(3)
pUCH2-8	Vector with hygromycin B (*hph*) resistance gene	(2)
pYYH3	*ThlaeA* deletion cassette in pUCH2-8	This study
pYWL156	Vector with hygromycin B resistance gene and *gpdA promoter*	This study
pYHL94	*gpdAP*::*ThlaeA*::*hph in* pYWL156	This study

Note: TXX = original transformant, pXX = plasmid

Table S2. Primers used in this study.

Primer name	Oligonucleotide sequence (5’-3’)	Uses
KOLaeA 5F F	CGAACCGGCAAGCTTATTAG	5 flanks amplification of LaeA deletion cassette
KOLaeA 5F R	ACCCTGGCGTTACCCAACTTAATCGCCTTGC<br>AGCACATCGAAGGATCCGAGAACTGAGG	
KOLaeA 3F F	GTGTGAAATTGTTATCCGCTCACAATTCCAC<br>ACAACATAGGAGCTTGAACTGCCTATGG	3 flanks amplification of LaeA deletion cassette
KOLaeA 3F R	GTGCTGCTTGGTGCTTCTG	
KOLaeA nest F	CTCTGCTGCTTGCATTGG	For Screening mutants
KOLaeA nest R	GTGACACCAGAGCACCTG	
LaeA RT F	CTTGCCTGGACTTCACTCC	For Screening mutants
LaeA RT R	CCAAGTGCTTGGAAGTTGC	
G418 SCR 3F F	CGTTACCCAACTTAATCGCC	For Screening mutants
G418 SCR 5F R	CACAGGAGGTACTAGACTACC	
OELaeA 5F F	GGATCCCCCGGGCTGCAGGAATTCGATATCA<br>CAATCCACCTTGCTCCGAC	5 flanks amplification of LaeA overexpression cassette
OELaeA 5F R	AACTGTGATAAACTACCGCATTAAAGCTGGC<br>GACTATGATGAGACCTTTG	
OELaeA 3F F	AGCTACCCCGCTTGAGCAGACATCACAAATG<br>TCTCGAAACGCTCGAAATG	5 flanks amplification of LaeA overexpression cassette
OELaeA 3F R	CTGGGTACCGGGCCCCCCCTCGAGGTCGACA<br>AGTGCTTGGAAGTTGCAC	
OELaeA SCR5F F	GTCCAGAGGTCGTCGTTTAG	For Screening mutants
OELaeA SCR3F F	GACAAGGTCGTTGCGTCAG	
hyg scr 5F R	GCCTATGCCTACAGCATCC	For Screening mutants
hyg scr 3F F	CTTGCTGCGGTGGATGGTTC	
OELaeA SCR3F R	GATGAGCACTGAGGCAAGC	For Screening mutants

Table S3. LaeA orthologues for phylogenetic tree in the given organisms and their conservative property with LaeA in *Aspergillus nidulans*.

**Fungal strains**	**GenBank accession number**	**Coverage / identity with *A. nidulans* (%)**
Alternaria alternata As-27	BAP58880.1	81/48.43
Aspergillus carbonarius ITEM 5010	OOF95411.1	95/77.99
Aspergillus fischeri NRRL 181	XP_001264291.1	99/74.53
Aspergillus flavus NRRL3357	B8N406.2	99/76.61
Aspergillus fumigatus	AAR01218.1	88/80.59
A. fumigatus Af293	XP_752835.1	99/73.99
Aspergillus fumisynnematus	A0A0H4LJX8.1	99/74.53
Aspergillus kawachii IFO 4308	GAA92888.1	72/80.74
Aspergillus nidulans FGSC A4	C8VQG9.1	100/100
A. nidulans	Q6TLK5.1	100/100
Aspergillus niger ATCC 1015	G3XRG4.1	99/73.92
A. niger CBS 513.88	XP_001389674.2	99/73.92
Aspergillus oryzae RIB 40	AB267276.1	99/76.61
Aspergillus pachycristatus NRRL 11440	QFQ50463.1	96/87.43
Aspergillus parasiticus NRRL 5862	AAX68414.1-2	99/76.61
Aspergillus terreus MUCL38669	ARB51348.1	99/73.66
Botrytis cinerea B05.10	XP_024548614.1	77/42.14
Chaetomium globosum CBS148.51	EAQ93455.1	59/37.87
Cochliobolus heterostrophus	AEP40318.1	75/51.2
Fusarium fujikuroi IMI 58289	S0DQI7.2	80/33.97
Fusarium graminearum PH-1	I1RAW4.2	79/34.2
Fusarium verticillioides 7600	XP_018742754.1	75/35.27
Monascus pilosus	A2SUH3.1	94/70.62
Monascus purpureus M1	QGA78456.1	94/70.62
Monascus ruber M7	AIY63188.1	94/70.62
Neurospora crassa OR74A	XP_011392800.1	81/42.21
Penicillium chrysogenum	ACD50375.1	95/61.23
Penicillium citrinum IMB002	ADL63139.1	95/61.76
Penicillium digitatum PHI26	EKV10385.1	88/63.14
Penicillium expansum MD-8	XP_016597816.1	95/62.83
Penicillium oxalicum 114-2	EPS25650.1	88/70.72
Pyricularia oryzae 70-75	XP_003714136.1	78/37.01
Trichoderma atroviride ATCC 74058	AGT59504.1	75/37.8
Trichoderma arundinaceum IBT 40837	RFU72149.1	81/36.19
Trichoderma citrinoviride TUCIM 6016	XP_024752164.1	73/35.92
Trichoderma guizhouense NJAU 4742	OPB44277.1	75/37.93
Trichoderma harzianum T6776	KKO98277.1	75/38.28
Trichoderma hypoxylon CGMCC 3.17906	AXL154_06481	84/36.3
T. hypoxylon CGMCC 3.17906	AXL154_06748	90/29.68
Trichoderma lentiforme CFAM-422	KAF3073341.1	73/37.1

Trichoderma longibrachiatum ATCC 18648	PTB77947.1	73/37.32
Trichoderma reesei	AFX86442.1	75/37.58
T. reesei QM6a	AFK30952.1	75/37.58

Table S4. LaeA orthologues in *Trichoderma* spp and their conservative property with LaeA in *T. atroviride* ATCC 74058 and *T. reesei* QM6a.

**Strains in *Trichoderma***	**GenBank accession number**	**Coverage / Identity (%) with LaeA in**	
		***T. atroviride* ATCC 74058**	***T. reesei* QM6a**
T. atroviride ATCC 74058	AGT59504.1	100/100	99/71.64
T. arundinaceum IBT 40837	RFU72149.1	99/70.4	100/71.71
T. citrinoviride TUCIM 6016	XP_024752164.1	95/70.45	100/94.28
T. guizhouense NJAU 4742	OPB44277.1	97/69.01	100/74.17
T. harzianum T6776	KKO98277.1	97/68.13	100/73.87
T. hypoxylon CGMCC 3.17906	(AXL154_06481)	99/71.55	100/77.11
T. hypoxylon CGMCC 3.17906	AXL154_06748	84/26.51	82/38.54
T. lentiforme CFAM-422	KAF3073341.1	95/68.36	100/75.08
T. longibrachiatum ATCC 18648	PTB77947.1	95/71.64	100/94.88
T. reesei	AFX86442.1	97/71.72	100/100
T. reesei QM6a	AFK30952.1	99/71.64	100/100

Supplementary Figures

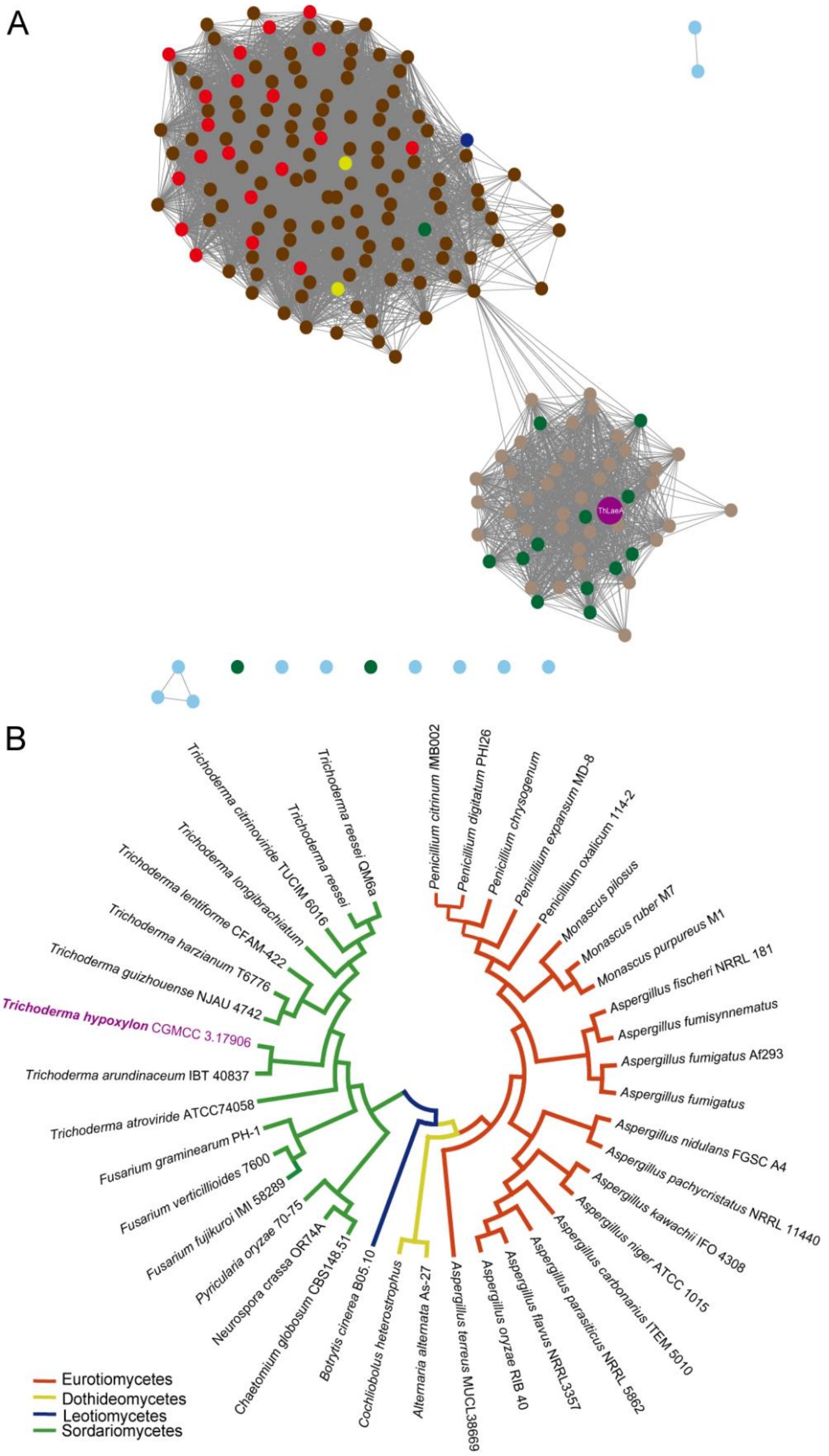

Figure S1. Phylogenetic tree analysis and sequence clustering of ThlaeA orthologues in fungi. (A) Sequence clustering of ThlaeA orthologues. (B) Phylogenetic tree of ThlaeA with known laeA-like regulators by maximum likelihood phylogeny analysis. Orthologues of LaeA in the fungal organisms were marked with different colors to recognize the taxa species.

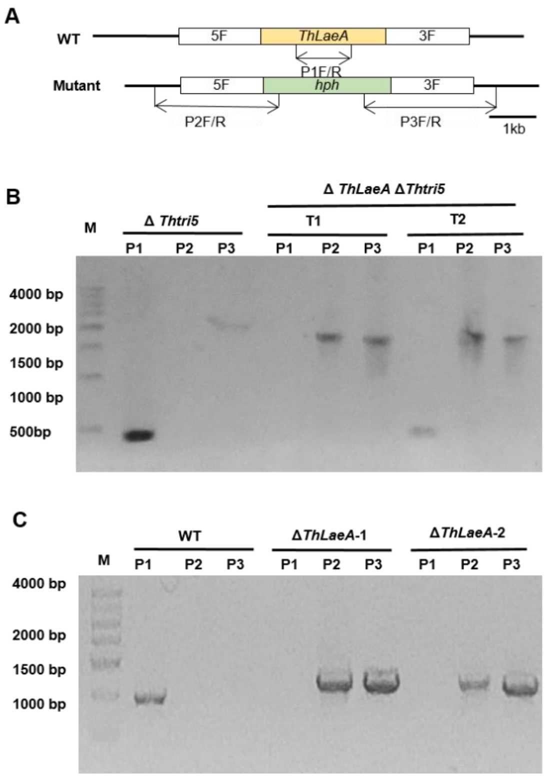

Figure S2. Generation of the *ThlaeA* deletion mutant strains in *T. hypoxylon* by diagnostic PCR. (A) Schematic illustration of disruption of *ThlaeA* and confirmation of Δ*ThlaeA* strains by diagnostic PCR. (B) Confirmation of Δ*ThlaeA* strains in Δ*Thtri5* host by diagnostic PCR. (C) Confirmation of Δ*ThlaeA* strains in *T. hypoxylon* wild type by diagnostic PCR. Three pairs of primers including P1F/R, P2F/R and P3F/R were used for mutant screening.

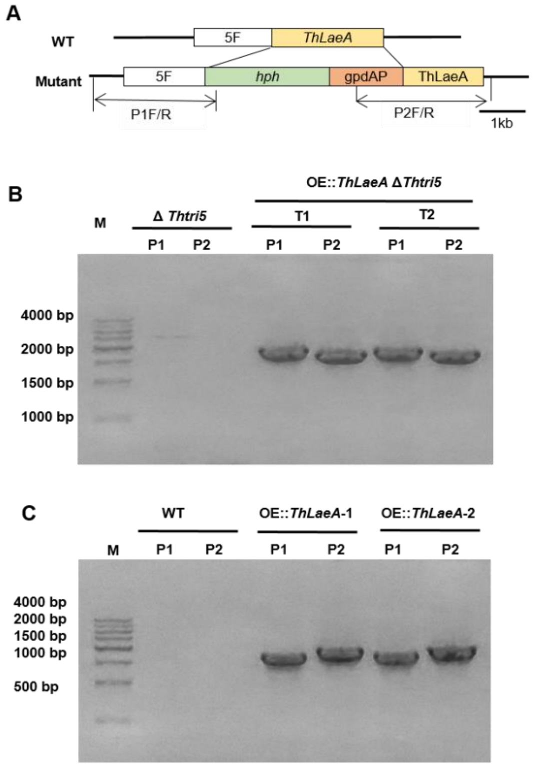

Figure S3. Generation of the OE::*ThlaeA* mutant strains in *T. hypoxylon* by diagnostic PCR. (A) Schematic illustration of overexpression of *ThlaeA*. (B) Confirmation of OE::*ThlaeA* strains in Δ*Thtri5* host by diagnostic PCR. (C) Confirmation of OE::*ThlaeA* strains in *T. hypoxylon* wild type by diagnostic PCR. Two pairs of primers including P1F/R and P2F/R were used for mutant screening. The specific bands of PCR were only found in mutants but not in control strain.

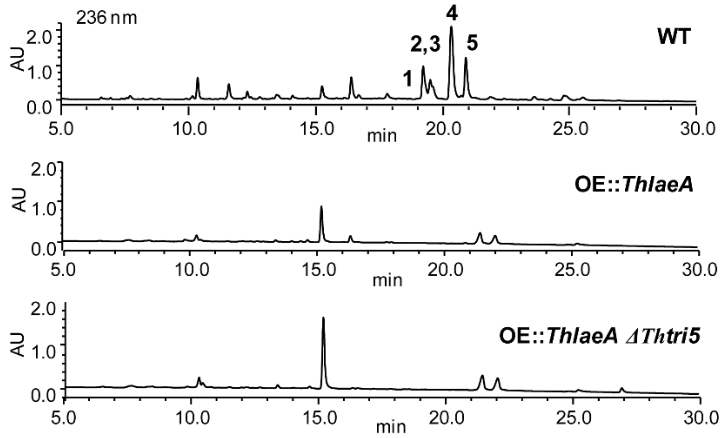

Figure S4. Metabolic profiling of HPLC analysis for OE::*ThlaeA* and OE::*ThlaeA*Δ*Thtri5* mutants compared with the wild-type strains in *T. hypoxylon*. UV absorptions at 236 nm are illustrated. **1-3**: tricinoloniol acids A-C. **4**: fusidilactone A. **5**: harzianum B.

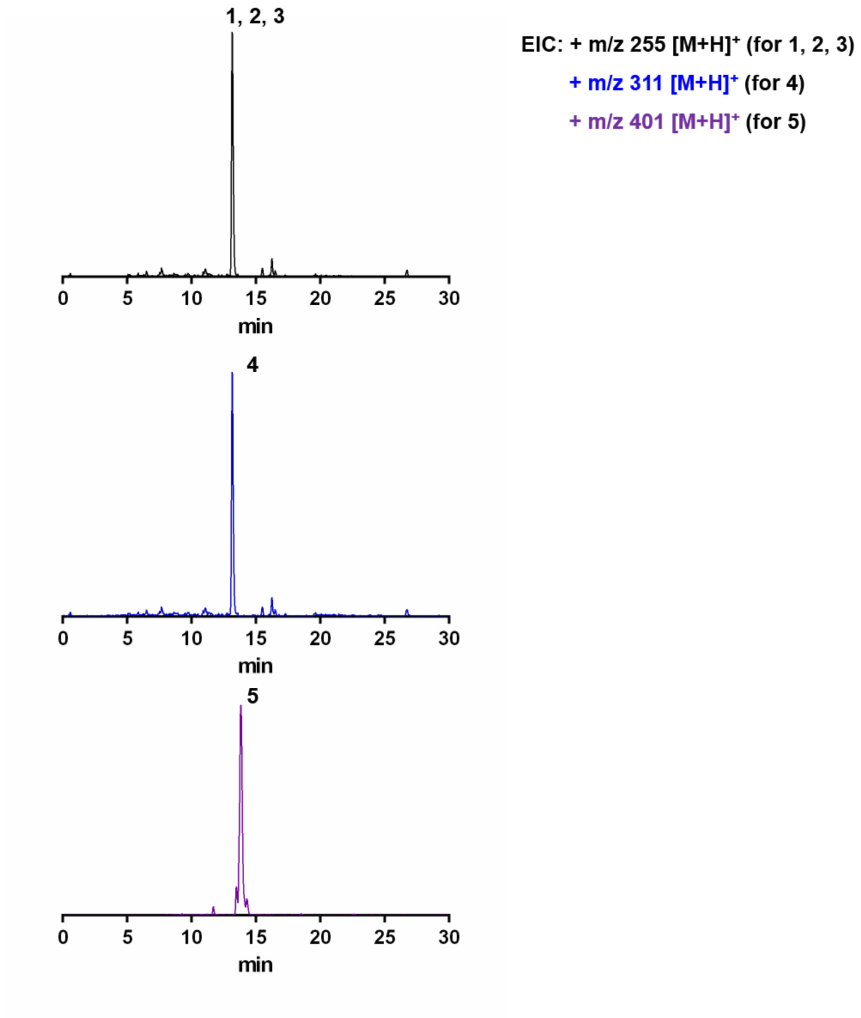

Figure S5. LC−MS analysis of the compounds **1**−**5** in *T. hypoxylon* wild-type. Note: a. MW of **1**−**3** were detected at 255; b. MW of **4** was detected at 311; c. MW of **5** was detected at 401.

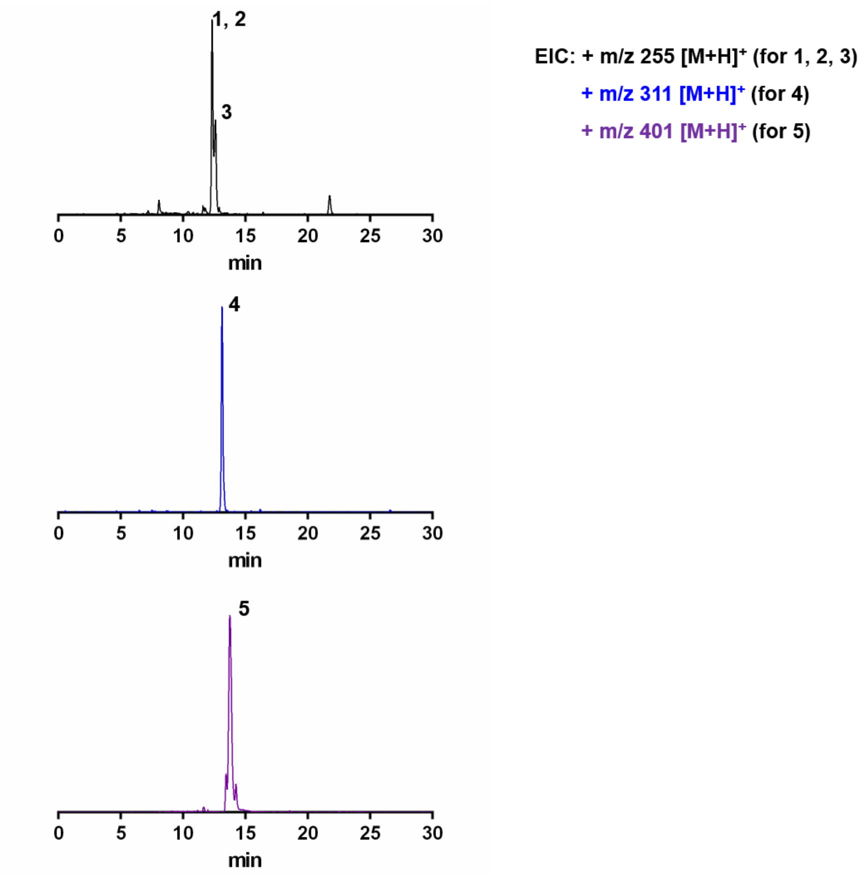

Figure S6. LC−MS analysis of the compounds **1−5** in TYHL14 (Δ*ThlaeA*). Note: a. MW of **1−3** were detected at 255; b. MW of **4** was detected at 311; c. MW of **5** was detected at 401.

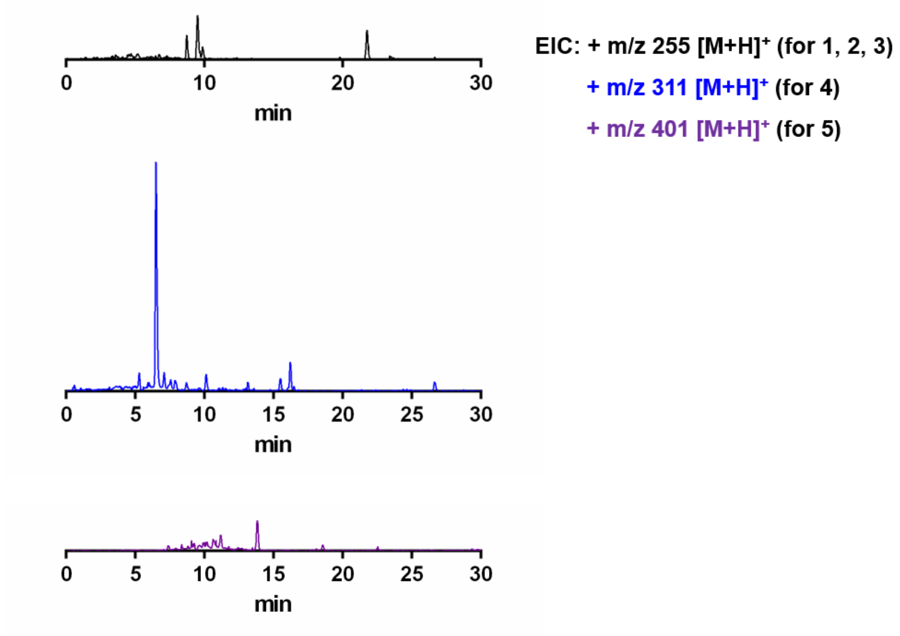

Figure S7. LC−MS analysis of the compounds **1**−**5** in TYHL26 (Δ*Thtri5*). Note: a. MW of **1**−**3** were not detected at 255; b. MW of **4** was not detected at 311 and at about 13 min; c. MW of **5** was not detected at 401.

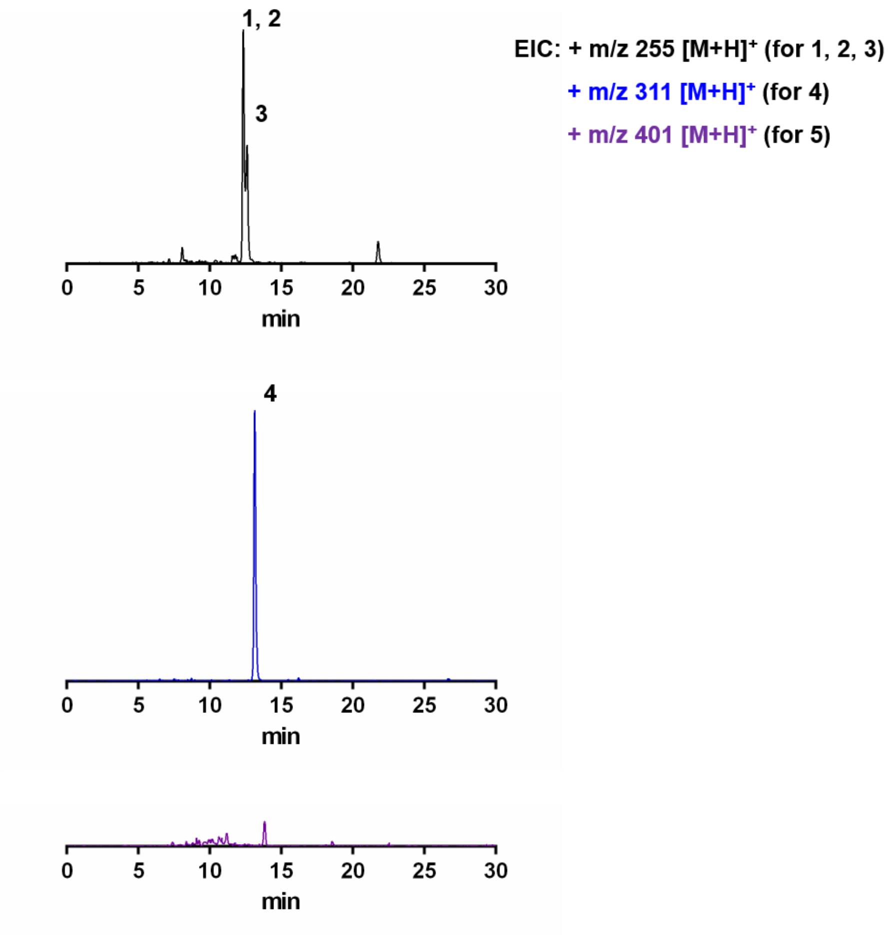

Figure S8. LC−MS analysis of the compounds **1−5** in TYHL49 (Δ*ThlaeA*Δ*Thtri5*). Note: a. MW of **1−3** were detected at 255; b. MW of **4** was detected at 311; c. MW of **5** was not detected at 401.

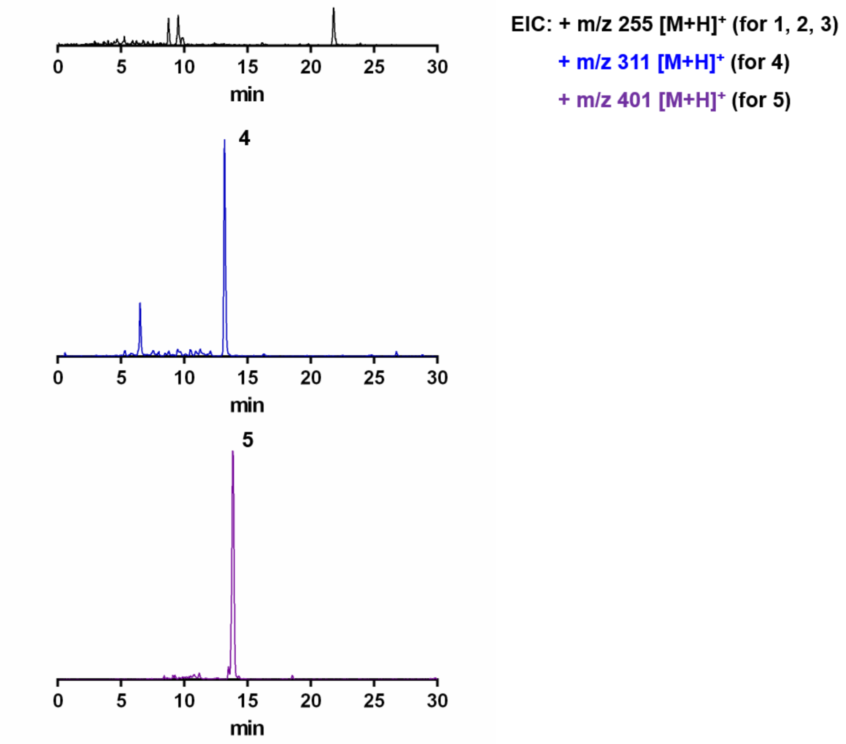

Figure S9. LC−MS analysis of the compounds **1**−**5** in TYHL55 (OE::*ThlaeA*). Note: a. MW of **1**−**3** were not detected at 255; b. MW of **4** was detected at 311; c. MW of **5** was detected at 401.

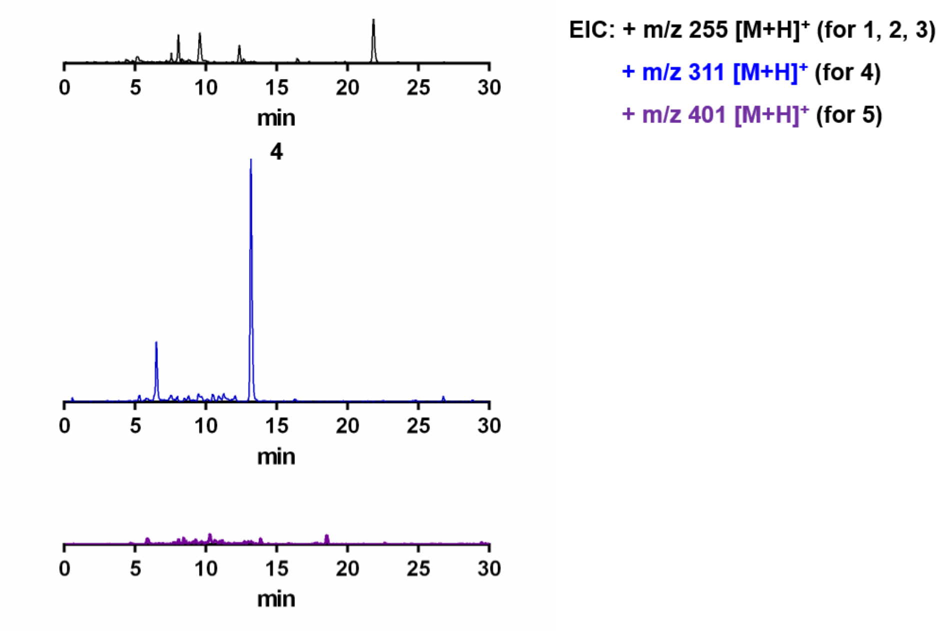

Figure S10. LC−MS analysis of the compounds **1−5** in TYHL56 (OE::*ThlaeA*Δ*Thtri5*). Note: a. MW of **1−3** were not detected at 255; b. MW of **4** was detected at 311; c. MW of **5** was not detected at 401.

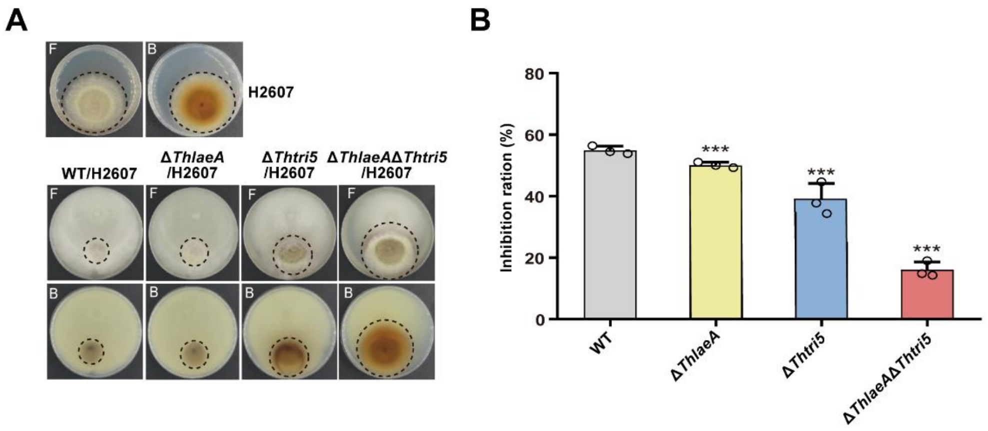

Figure S11. Antagonistic activity of *T. hypoxylon* and its mutants against the pathogenic fungus *Hypoxylon* sp. H2607. (A) Phenotypes of *T. hypoxylon* and its mutants in against *Hypoxylon* sp. H2607. (B) The inhibition ratio of the test strains against the *Hypoxylon* sp. H2607. All strains were grown on PDA at 25 ℃ for 4 days. All error bars are expressed as mean ± SD. Statistical analysis was performed by using One-way ANOVA (Significant ***$p < 0.001$).

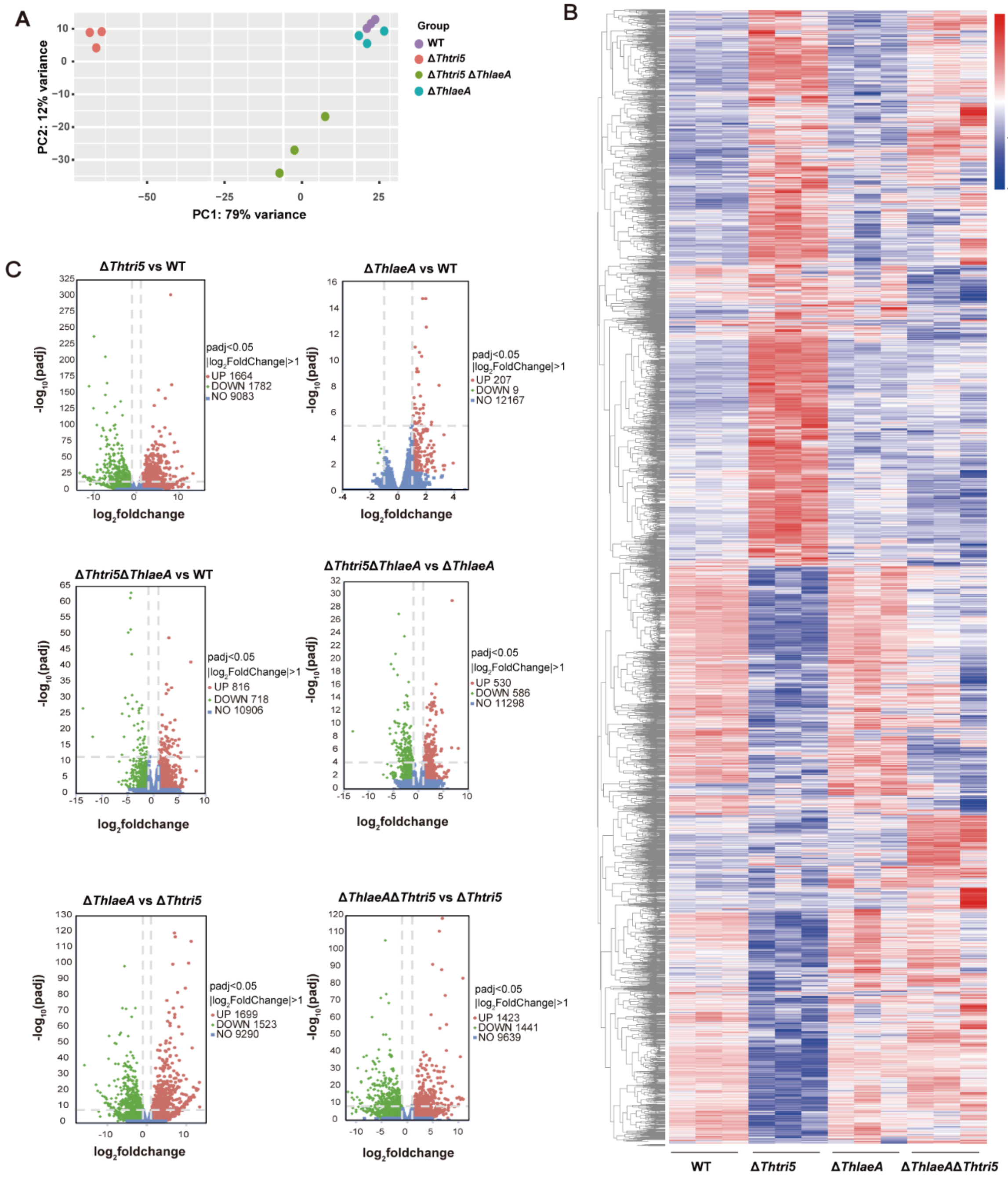

Figure S12. Transcriptome-wide analyses of differentially expressed genes in the Δ*Thtri5* and Δ*ThlaeA* mutants versus the control in *T. hypoxylon*. (A) Principal component analysis of gene expression in T. *hypoxylon* and its mutants. (B) Heatmap showing differentially expressed genes of Δ*Thtri5*, Δ*ThlaeA* and Δ*ThlaeA*Δ*Thtri5* mutants compared to the control. (C) The volcano plots show the differentially expressed genes of ΔThtri5, Δ*ThlaeA* and Δ*ThlaeA*Δ*Thtri5* mutants compared to the control.Differentially expressed genes: padj < 0.05, |Log_2foldchange| > 1.

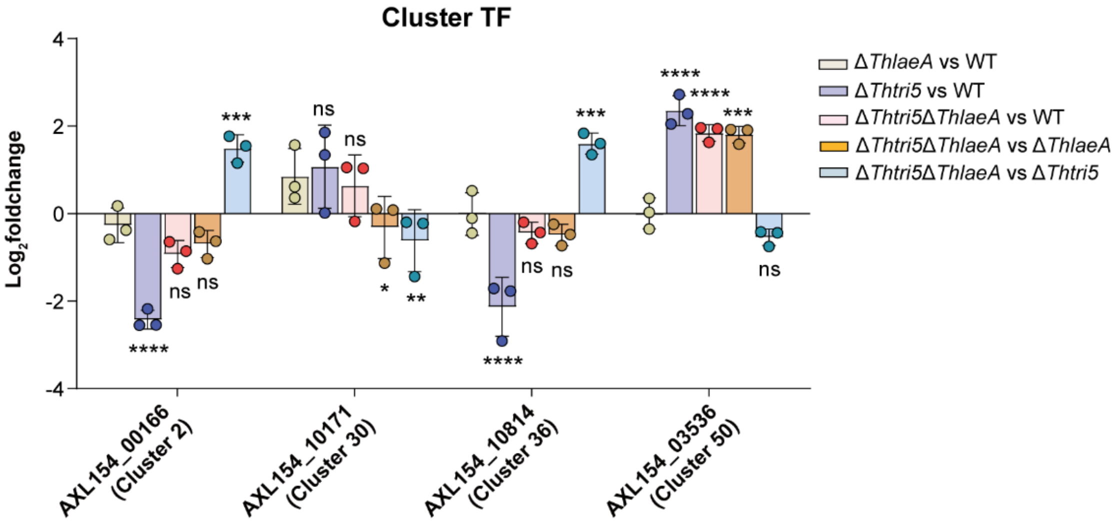

Figure S13. Transcriptome analysis of intra-cluster transcription factor (TF)-related genes in Δ*Thtri5*, Δ*ThlaeA* and Δ*ThlaeA*Δ*Thtri5* mutants compared to the control.

Supplementary References

1. Sun J, Pei Y, Li E, Li W, Hyde KD, Yin WB, Liu X. 2016. A new species of Trichoderma hypoxylon harbours abundant secondary metabolites. Sci Rep 6:37369.
2. Liu H, Wang G, Li W, Liu X, Li E, Yin WB. 2018. A highly efficient genetic system for the identification of a harzianum B biosynthetic gene cluster in *Trichoderma hypoxylon*. Microbiology 164:769-778.
3. Pu Y, Liu H, Chen G, Guo L, Yin W. 2020. Identification and functional study of NRPS1 in *Trichoderma hypoxylon*. Acta Microbiologica Sinica 60:2350–2361.
